# The Efficacy of Treadmill Training on Walking and Quality of Life of Adults with Spastic Cerebral Palsy: A Randomized Controlled Trial

**Published:** 2019

**Authors:** Fariba BAHRAMI, Shohreh NOORIZADEH DEHKORDI, Mehdi DADGOO

**Affiliations:** 1 Department of Physiotherapy, School of Rehabilitation Sciences, Iran University of Medical Sciences, Tehran, Iran

**Keywords:** Cerebral palsy, Adult, Treadmill training, Walking, Quality of life

## Abstract

**Objectives:**

We aimed to evaluate the efficacy of treadmill training on walking speed and endurance and quality of life in ambulatory adults with spastic cerebral palsy (CP) versus traditional physiotherapy.

**Materials & Methods:**

Participants (17 men, 13 women; mean (SD) age 25 yr, 9 m (7 yr, 10m) range 18-45) with Gross Motor Function Classification System (GMFCS) levels below IV (I, II, and III) from the Ra’ad Rehabilitation Goodwill Complex, Tehran, Iran randomly were allocated to the experimental and the control groups each with 15 persons in 2014. The training (treadmill for experimental group and conventional physiotherapy for control group) was conducted two times a week for 8 weeks. Statistical analysis was made by Repeated Measures of ANOVA for changes within the group during the time and Independent *t* and Mann-Whitney U tests for the differences between the groups.

**Results:**

Although the experimental group showed a significant improve in the gait speed [1.08(0.47) m/s to 1.22(0.50) m/s] (*P*=0.002) and in the gait endurance [291.13(160.28) m to 342.63 (174.62) m] (*P*=0.002), however the changes of the outcome measures of walking and quality of life the between groups were not significant.

**Conclusion:**

The treadmill training without body weight support would improve walking speed and endurance in adults with spastic CP. It would not be however more effective than the traditional physiotherapy to increase the gait performances and function and the quality of life in adults with CP.

## Introduction

Cerebral palsy (CP) is the result of damage to the brain from birth to two years old. However, the clinical signs include spasticity, pain and stiffness, declined mobility and function and gait impairments are deteriorated by aging (1). Overall, 44% of the adults with CP experience deterioration of walking skills before age 35 (2).

The causes of impaired mobility and function in CP patients consists of abnormal walking patterns, spasticity, stiffness, muscle weakness, reduced walking speed and endurance and immobility (3-5). In adults with CP, limited activities and levels of participation including the social interaction, employment, marriage, education and hobbies in society would reduce their quality of life (6, 7). Services provided for adults with physical disabilities are often inadequate, even in developed countries (8). At least, the obvious low preference given to the adults with CP is partially, because the physiotherapists recognized that their attempts are better applied to the young child (9)

Treadmill training is one of the functional physical therapy practices based on the task-oriented approaches (10), to improve the gait impairments and also the participation of the patients with neurological disabilities (11-13). Many types of research have demonstrated the effectiveness of the treadmill training with or without partial body weight support in children with CP to improve the gross motor function, and to enhance the lower extremity muscle strength, walking speed and endurance, as well as the Spatio-temporal parameters of the gait, and balance and quality of the life (14-16).

A randomized controlled clinical trial (17) compared the effects of treadmill training and training with overground walking (both without partial weight support) on motor skills of thirty-six children with CP (age range of 3-12 yr; levels I-III of the Gross Motor Functional Classification System). The schedule succeeded seven consecutive weeks with two sessions per week, following with the next four weeks of follow-up. Treadmill training is more efficient, regarding functional mobility, functional performance, gross motor function and functional balance, than the training with overground walking in the children with CP.

There was a randomized controlled trial (18) on 22 adolescents (13-19 yr old) with CP. The experimental group received treadmill training without body weight support (with comfortable speed) and the control group was treated with conventional physiotherapy (three sets of exercises with mat activities, functional gross motor activities, balance, and gait training). The program lasted 12 wk with a frequency of three times per week for both groups. The spasticity, self-selected walking speed, and gross motor function were measured before and after the training. The treadmill training without body weight support may have positive effects on improving the gross motor function and walking speed of the adolescents with spastic CP, without negative effects on spasticity. 

Evidence for the effect of physiotherapy on adolescents and adults with CP is sparse, and therefore there is a need for well-designed physiotherapeutic trials for these people (19). There is no study yet on the effectiveness of treadmill training on the adults over 20 yr old with spastic CP. The aim of this research was to determine the success of the treadmill training on the gait speed and endurance and quality of life of the adults with CP and to compare it with the traditional physiotherapy in a randomized controlled trial. 

**Figure 1:**
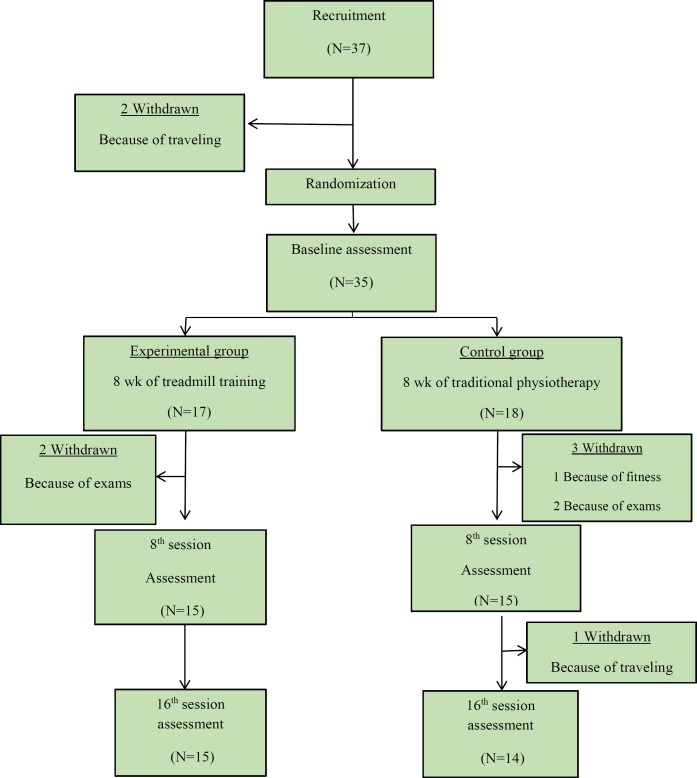
Flow chart of progression of the study

**Table 1:** Characteristics of adults with spastic cerebral palsy

Variable	Experimental group	Control group	Total
GMFCS(I/II/III)^*^	7/2/6	8/2/5	15/4/11
Gender(male/female)^*^	8/7	9/6	17/13
Type(h/d/q)^*^	7/6/2	7/7/1	14/13/3
Age(years)^**^	25.9(7.7)	25.1(4.3)	25.4(6.22)
Weight(Kg)^**^	56.1(11.05)	60.3(13.2)	58.2(1.2)
Height(cm)^**^	159(10.6)	160(10.09)	160(10.2)
10MWT^**^	1.08(0.47)	0.99(0.56)	1.04(0.51)
6MWT^**^	291(160)	279(161)	285.41(158.30)
WHOQOL-Brief^**^	3.55(0.55)	3.31(0.68)	3.43(0.62)

Note: Values are mean (SD)^**^ or number^*^

GMFCS: Gross Motor Function Classification System. I & II refers to mild and III refers to moderate severity of CP.

Types of spastic CP: h indicates hemiplegia, d indicates diplegia, q indicates quadriplegia.

10 MWT: 10 m walk test

6 MWT: 6 min walk test

WHOQOL-Brief: Quality of Life

**Table 2:** Results of repeated measures of ANOVA

	^*^10 MWT		^*^6 MWT		^*^ QOL	
	experimental	control	experimental	control	experimental	control
^*^SS	.15	.00	19891.8	7377.5	.09	.37
^*^df	1	1	1	1	1	1
F	14.11	.44	13.58	5.75	1.15	3.06
Sig.	**.00**	.51	**.00**	**.03**	.30	.10

^*^SS: type III Sum of Squares

^*^df: degrees of freedom

^*^10 MWT: 10 m walk test

^*^6 MWT: 6 min walk test

^*^WHOQOL-Brief: Quality of Life

**Table 3:** Group outcome measure scores and between-group significance

Outcome Measures	Pre		Middle		Post		*P-*value
	Experimental N=(17)	Control N=(18)	Experimental N=(15)	Control N=(15)	Experimental N=(15)	Control N=(14)	
10 MWT	1.08(0.47)	0.99(0.56)	1.14(0.51)	1.03(0.56)	1.22(0.50)	1.02(0.61)	0.42
6 MWT	291.13(160.28)	276.10(167.19)	330.13(176.29)	293.85(178.55)	342.63(174.62)	308.57(181.22)	0.61
QOL	3.55(0.55)	3.33(0.69)	3.69(0.57)	3.35(0.76)	3.66(0.59)	3.57(0.67)	0.69

WHOQOL-Brief: Quality of Life

**Figure 2:**
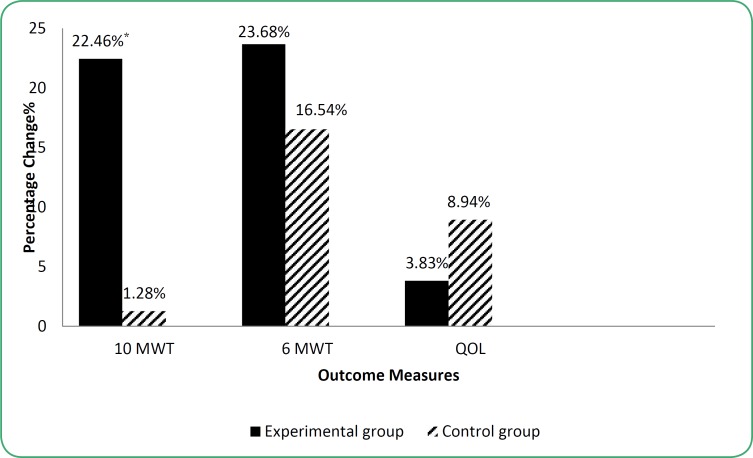
Comparison of mean percentage change scores of the outcome measures of experimental and control groups from pre to post- introduction assessments

* Statistically significant (*P*<0.05) difference from control group

10 MWT: 10 m walk test

6 MWT: 6 min walk test

WHOQOL-Brief: Quality of Life

## Materials & Methods

**Participants**

The participants were recruited from the Ra’ad Rehabilitation Goodwill Complex, an educational and rehabilitation charity center in the northwest of Tehran, Iran in 2014. The participants were included whose spastic CP was approved by a neurologist, aged between 18-45 yr and had ability to walk with or without auxiliary facilities according to the level I to III of the Gross Motor Function Classification System (GMFCS). This refers to mild to moderate severity of CP so that they would walk with or without the assistive devices (20). They had ability to follow commands and they got a minimum score of 18 from the Mini-Mental State Examination (MMSE) (21). If the people had surgery on the lower limbs or had taken any oral or injectable anti-spasm medicine during the last 6 months, or they had other concurrent orthopedic or neurologic diseases such as Multiple Sclerosis, Alzheimer's, Parkinson’s, fractures and soft tissue injuries in the lower limbs, had heart disease or uncontrolled epilepsy, they were excluded from the study.

**Apparatus**

A motorized treadmill (G Series of Tunturi) with a minimum speed of 0.5 km per hour and capability to display the distance in kilometer and the walking speed in kilometer per hour was used in this trial. 

**Ethical approval**

After permission from the Research Ethics Committee with license no. 94/105/58 and registration of clinical trial issue with no.IRCT2014022216680N1 from the Iran University of Medical Sciences, the informed consent was obtained from the participants after full description of objectives and methods of the project. 

**Procedure**

The demographic data and personal information were collected from the patients’ records archived in the physiotherapy clinic of the institution. Before group random allocation, two people from 37 participants were excluded because of traveling, so the number of volunteers diminished to 35.

The participants were stratified according to the severity of the disorder (level I-II or level III of GMFCS), kinds of CP (hemiplegia, diplegia or quadriplegia) and sexuality (male or female) and then randomly allocated into two groups. This process was led by physiotherapists that worked in the institution and were blinded to the intervention. To assign the participants to the experimental or control groups, sealed envelopes were used. Eighteen individuals allocated to the experimental training group and seventeen people to the control training group.

The protocol employed in the present study for experimental and control groups, was two sessions per week for 8 consecutive weeks that each lasted forty minutes (excluding the rest time). The experienced physiotherapists in treating CP conducted all training sessions.

The treatments for the experimental group consisted of static stretching for shortened muscles of lower limbs for 5 min and then stationary biking with moderate resistance for another five minutes for warm-up. Then they walked on the treadmill for thirty minutes, which divided to three times 10 min with two minutes of rest intervals. People started to walk at speed of 0.5 km/h. The speed of the treadmill could be added depending on each patient’s tolerance every session. In subsequent session they began to walk with maximum speed rate gained in the previous session. Over time, the rest period of treadmill training declined, so that in the last three sessions of treatment, people walked on the treadmill uninterrupted for thirty minutes.

 The treadmill had parallel bars and participants took the bars to prevent falling. For correction of the gait pattern by visual feedback, a mirror was placed in front of the treadmill and verbal commands were given. Heart rate and oxygen saturation were measured before and after the treadmill training with a pulse oximeter (wellkong Inc. British). In this case the dominant hand was placed on a fixed plate. If a person's heart rate increased to 60% to 75% of maximum heart rate (220-age), the rest time was added and the speed would not be increased. It happened twice for one of the volunteers during the trial. 

The volunteers were asked to wear their usual footwear (including orthopedic shoes or brace) during therapy sessions and also in regular assessments. Specifications about each candidate, including their footwear, heart rate at the beginning and end of the treadmill training, the break time duration, and the speed and the distance walked on the treadmill were recorded in a notebook. 

The control group was treated with traditional physiotherapy. This included the static stretching of shortened muscles of lower extremities for five minutes, sport biking with moderate resistance for five minutes, strengthening exercises (using weights cuffs) for extensor and abductor muscles of the hip and knee joints (antispastic muscles) for 15 min and final 15 min of balance exercises and PNF (Proprioceptive Neuromuscular Facilitation) exercises. PNF consisted of the mobility, stability and controlled mobility techniques in quadruped, kneeling and standing positions which totally lasted for 40 min. At each session, the repetition of strengthening exercises was increased depending on patient tolerance. Participants were asked not to participate in any other rehabilitation or sports programs during the intervention 

**Outcome Measures**

Two experienced physiotherapists in assessing movement disorders, other than who provided the treatment, conducted the evaluations of gait performance and quality of life questionnaire three times (at baseline, in the ninth session and in the sixteenth session). 

The order of the assessments was random and implemented between 10:00 and 12:00 in the morning. The 10-Meter Walk Test (10MWT) with fastest speed was used to measure gait speed. This test is functional, reliable, easy, safe and inexpensive to administer (22). Before the trial, inter and intra rater reliability of the 10 MWT evaluated on 30 adults with CP within a week. The results indicated excellent reliability of the 10 MWT among adults with spastic CP [Standard error of measurement (SEM) <0.07, Intraclass correlation coefficient (ICC)>0.98]. To conduct the test a mid-stretch of at least 14 m length corridor was set for the walking course. The start and stop lines with two meters plus at the beginning and end of the course were considered to remove acceleration or deceleration. Before the test, the subjects were warned not to run. Participants were asked to walk with their fastest speed. After about 10 min of rest, the next test was conducted.

The 6 Minute Walk Test (6 MWT) was used to measure walking endurance. This test has high test-retest reliability (ICC=0.98, 95% confidence interval (CI) lower bound>0.64) in 31 ambulatory children with spastic CP (5). The walking course was set in a corridor with length of 20 m. Adults were instructed to walk with their maximum speed (not to run). The blood pressure was measured before and after testing to ensure adults’ health. Individuals, who used the assistive device for walking, were asked to utilize the same device during the tests.

In this study, changes in the quality of life of the participants were assessed by the World Health Organization Quality of Life Questionnaire- Brief (WHOQOL-BREF). The WHOQOL-BREF, an abbreviated 26 item version of the WHOQOL-100, was developed using data from the field-trial version of the WHOQOL-100. This questionnaire is a generic tool for measuring improvement in the quality of life-related to health care. Both the WHOQOL-100 and the WHOQOL-BREF have been shown to display good discriminant validity, content validity, and test-retest reliability. Domain scores produced by the WHOQOL-BREF have been shown to correlate at around 0.9 with The WHOQOL-100 domain scores. The Cronbach alphas demonstrate good internal consistency for the facets with a range of 0.65 to 0.93 (23). It demonstrated to have a good to excellent reliability and an acceptable validity in Iran (24). The WHOQOL-BREF questionnaire measures physical health, psychological, social relationships and environmental domains. 

**Statistical Analysis**

Based on the literature with similar outcome measure (25), with the effect size (Cohen d= 1.02) and the sample size of 8 participants per group, there would be an 80% power to detect between-group changes at *P*<.05. Because of the age differences of our trial and the possibility of drop-outs, more participants screened. People, who participated in less than 30% of training sessions, were excluded from the statistical analysis.

Analyses were performed using SPSS software ver. 16 (Chicago, IL, USA). To evaluate the normal distribution of numerical variables, the Kolmogorov-Smirnov (KS) test was used. The independent *t*-test was used to determine the equality of the data of the groups before the intervention. To investigate the changes in walking speed, walking endurance and quality of life of each group as a factor during the time, a general linear model of repeated measure analysis of variance (ANOVA) was used. When we got a significant F-test result, Then Bonferroni utilized as a post hoc test to specify which time shows significant change from the test. Independent *t* or Mann-Whitney U tests, as appropriate, were used to comparison between-group change scores. We utilized the percentage change scores for further evaluations. Significance was set to 0.05 for all analyses. 

## Results

Of 35 participants screened and assessed at baseline, 5 patients before the second assessment withdrew from the study because of traveling. The progression of participation through study process is summarized in Figure 1. There were no significant differences between the two groups for demographic characteristics (age, weight and height) or outcome measures at baseline (*P*<0.05) (Table 1). 

Of 16 sessions, attending sessions for the control group was 11/8± 2/9 with a minimum of 9 and a maximum of 16, and for the experimental group was 13/9 ± 1/6 with a minimum of 11 and maximum of 16 sessions for two months. No injury involved to anyone in the study due to interventions. 

As training intensity increased in control group by adding the repetition of constant weight exercise (from 10 repetitions in the first session to 30 repetitions in the last session), the speed of treadmill and distance walked on treadmill increased in the experimental group significantly (*P*=0.00). The speed of treadmill in Km/h progressed from 1.17±0.39 (Min-Max, 0.5-1.9) in the first session to 2.7±1.56 (Min-Max, 1-5.9) in the last one, and the distance walked on treadmill in Km increased from 0.5±0.27 (Min-Max, 0.16-0.98) in the first session to 1.23±0.65 (Min-Max, 0.3-2.5) in the last session. 

The Walking performances of experimental group significantly improved. The walking speed after two months [F(1, 14)=14.11, *P*=0.00] and the walking endurance after a month of training [F(1, 14)=13.58, *P*=0.00] were increased. There was no significant change in walking speed of control group, but walking endurance after two months of treatment was significantly increased [F(1,13)=5.75, *P*=.03]. The quality of life of each group was not changed over time significantly (Table 2). Between groups difference in any of the outcome measures was not significant (Table 3). 

## Discussion

This study showed that treadmill training improves walking speed and endurance of adults with spastic CP. Group was not a significant factor for all outcomes, while time effect was significant for speed after 8 wk, and for endurance after 4 weeks. Although there was no priority of treadmill training to regular physiotherapy for improving walking performances, only the treadmill group displayed a clinically meaningful improvement in walking speed of greater than 0.1 m/s (26). Increasing of walking speed in adults not only improves community ambulation and social interaction but also decreases fear of falling (27, 28). This result could perhaps be attributed to improvement of motor control as a result of reducing reaction time, reduction of the double support phase time of the gait and increasing the power of the lower body muscles and the performance of cardio-respiratory system (14, 29). Furthermore, self-reports of the participants on effectiveness of treadmill training indicated that their motivation to continue treatment and gait independence increased and morning stiffness and the fear of falling reduced.

Our findings of no effect of group allocation on any outcome measures are similar to another study (15) on comparing the effect of a supported speed treadmill training with strengthening exercise on 26 children with spastic CP. They reported improve in gait speed regardless of intervention group. Although they used supported speed treadmill training for 12 wk intensively and three-dimensional motion analysis as gait speed measure. Moreover, a randomized controlled trial (25), on 30 children with CP showed that partial body weight supported treadmill training and overground walking increased gait speed and endurance by week 4 and improvement continued to week 8 with no priority between groups. The training protocol was two times 30 min sessions of walking training per week for 8 wk, progressed as tolerated. 

In the partially similar findings of a randomized controlled trial (18) on adolescents with CP which both training groups showed increase in gait velocity but the differences of self-selected walking speed between groups was significant. Similar to our study, treadmill training without body weight support used for the experimental training group and the control group was given conventional physiotherapy. Perhaps lower age of participants in their study and longer of their trial (12 vs 8 wk) would have made this difference in the result.

The mean percent of change of walking speed of experimental group in our trial was 22.5% after intervention (Figure 2). This finding was smaller than the magnitude of change of 68% obtained in a study (16), which included school-aged children with moderate to severe functional disability. Children with relatively severe walking disabilities can make appreciable changes in performance after participation in a Partial Body Weight Supported Treadmill Training program. This is a logical consequence because the participants in this study were younger than our study.

Another finding of our study was improvement of gait endurance after one month of training for treadmill group and after two months for the control group. Perhaps treadmill training can increase gait endurance earlier. 

In the study of treadmill training without partial body weight support versus overground walking, the treadmill group obtained more increase in gait endurance (17). Probably getting more change in gait endurance of the experimental group versus the control group than present study, in addition to the age differences of both trial, is probably due to the training velocity that in their research was based on the cardiopulmonary effort test and gait training was performed at the aerobic threshold.

The result of a research (30) was partially contrary to the findings of our study. In this randomized controlled trial, treadmill training with partial body weight support was not only more efficient for improving walking speed and walking endurance than overground walking, but also the control group got more increase in the gait endurance than the experimental group. The differences of the severity of disability of the participants in the two studies, using the partial body weight support for experimental group and walking on the ground for control group, less speed and distance of treadmill than our training and younger participants in their research could cause different results between these two studies.

Another result of present study was no change in the quality of life in both experimental and control groups. Although, traditional physiotherapy and treadmill training improve walking performance of adults with spastic CP, but perhaps could not make a change in the quality of life of the participants in the study. 

Quality of life is a multidimensional concept that would cover all aspects of the physical, mental, psychological and environmental factors of life. Especially when our target population is adults with CP, to improve the quality of life beside the rehabilitation treatment, the psychological counseling and the work assistance should help to improve the employment, livelihood and family benefits of the participants. 

There are some studies (31, 32) that have similar results to our recent findings. They were conducted in adults with CP and the results of them negate a significant relationship between physical activity and functional gait status with the quality of life. The results studies on the treadmill effect on the quality of life of children with CP are in contrast with the result of our study (33, 34). Treadmill training can improve the social health and mental health domains of quality of life in children with CP. The differences in participant’s age, sample size, and type of protocol could be the causes of discrepancy of the result of our studies. 

In our knowledge this study is the first one to investigate the effect of the treadmill training on this age group of CP. This study showed that adults with CP would improve in the gait even slightly, so we suggest to the therapist who is working with CP and insurance companies do not omit elder CP. Our study is limited by lack of follow-up assessment after the end of the interventions and also absence of no-treatment control group to better judgment, especially for better comparison of the quality of life of adult participants. For qualifying the quality of life, this sample size of participants would be inadequate and with larger number of volunteers, we may obtain another result in the quality of life. Despite all the efforts were made we could not collect equal number of participants with every three groups of intensity of the CP from mild to moderate. This made a large variety of scores that made a problem in analyses.

We recommend future studies on CP that is better done on a particular group in term of severity of illness and other types of CP-like athetoid or ataxic rather than spastic CP. We recommend more studies to be carried out on rehabilitation interventions on adults with CP.

**In Conclusion**, treadmill training without body weight support would improve walking speed and endurance in adults with spastic CP. Although it would be no more effective than traditional physiotherapy to increase gait performances and function and quality of life in adults with CP.
